# Role of hormone replacement therapy in relieving oral dryness symptoms in postmenopausal women: a case control study

**DOI:** 10.1186/s12903-021-01966-6

**Published:** 2021-12-03

**Authors:** Lina Wang, Lilei Zhu, Yao Yao, Yajuan Ren, Hongcan Zhang

**Affiliations:** 1 Department of Gynecology, Shijiazhuang Maternity Hospital/Shijiazhuang Fourth Hospital, Shijiazhuang, 050000 Hebei China; 2 Department of Periodontology and Oral Medicine, Changsha Stomatological Hospital, Changsha, 41005 Hunan China; 3 Department of Orthodontics, Changsha Stomatological Hospital, Changsha, 41005 Hunan China; 4 Departments of Gynaecology and Obstetrics, Sichuan Integrative Medicine Hospital, Chengdu, 610041 Sichuan China; 5 Outpatient Clinic of Ethnomedicine, Youjiang Medical University for Nationalities, Baise, 533000 Guangxi Zhuang Autonomous Region China

**Keywords:** Postmenopausal females, Estradiol, Hormone replacement therapy, Xerostomia

## Abstract

**Background:**

To evaluate the efficacy of hormone replacement therapy in relieving oral symptoms in postmenopausal women presenting with genitourinary symptoms along with oral dryness.

**Methods:**

A case–control study was conducted after selecting 60 postmenopausal women. Oral dryness status of all the patients was evaluated with the help of questionnaire related to oral dryness. These subjects were divided into case group and control group on the basis of response to questionnaire of oral dryness. Unstimulated saliva samples were obtained and analyzed for estimation of salivary estradiol levels by enzyme linked immune sorbent assay technique. After analyzing the result of salivary estradiol levels, case group was subjected to hormone replacement therapy (HRT). The patients were followed up for their response towards oral dryness as well as salivary estradiol levels after the therapy.

**Results:**

The mean salivary estradiol level before HRT was significantly more among control group as compared to case group (*p* value < 0.001). Most of the patients complained of dry mouth (26 out of 30); reduced amount of saliva in the mouth (25 out of 30); dry mouth at night (28 out of 30); dry mouth during the day (25 out of 30) before HRT. These complains were significantly reduced after the therapy. The mean salivary estradiol in the case group levels increased significantly after HRT (*p* value < 0.001).

**Conclusion:**

The salivary estradiol levels were reduced in post menopausal women with the complain of xerostomia as compared to those without the complain of xerostomia. Further these levels can be recovered with the help of hormone replacement therapy.

## Background

Menopause is a physiological process occurring in women during fifth decade of life. As per the world health organization (WHO), it is characterized by permanent cessation of menstruation [1, 2]. It is attributed to loss of ovarian follicular activity which leads to reduced production of estrogen by the ovaries of such women [1–4]. Literature reveals that menopause is designated once there is interruption of menstruation in such woman for more than 12 months [5, 6]. The age of occurrence of menopause is considered to be multifactorial. Certain factors which can lead to an earlier menopause include genetic predisposition, high socioeconomic status, low parity and habits like smoking [7–10].

Menopause resulting in reduced estrogen is associated with various effects on the general health and well being of women. These include vasomotor symptoms, urinary symptoms, mood disturbances, sexual dysfunction and cognitive effects [11–13]. Literature also reveals that estrogen deficiency is associated with marked effects on the oral cavity due to reduction in the salivary flow which subsequently results in xerostomia [5, 7].

It is a well known fact that saliva is essential for maintaining the oral health of an individual. Estrogen deficiency can lead to reduction in the salivary flow leading to perception of dry mouth. However at least 40–50% reduction in the salivary flow is required to make the patient symptomatic and prone to develop xerostomia [5, 8–10]. This oral dryness can affect the quality of life of an individual as it interferes with chewing, swallowing and speech. It can also accelerate infection, caries and gum disease [5, 11].

Oral discomfort is increased among post menopausal women due to hormonal modifications [13]. In this era of advanced science and technology, life expectancy of each and every individual is raised [12–14]. Yalcin et al. [12] in the year 2005 revealed that most of the women have to spend one third of their life as a post menopausal women. Henceforth this becomes a matter of concern to understand the physiology behind the oral discomfort happening to the postmenopausal females. Some authors reveal that this oral discomfort can be reduced with the help of administration of HRT [14–17].

In a nutshell, oral health of postmenopausal women is compromised. A part of the fact that this high frequency of complications lead to reduced quality of life in such females, there is paucity of research correlating the effect of hormone replacement therapy in postmenopausal women experiencing xerostomia. Henceforth the aim of this present study was to evaluate the effect of HRT on oral dryness in such women. The hypothesis was HRT can recover the decreased salivary estradiol levels in post menopausal women.

## Methods

### Study design and study population

The study population consisted of post menopausal women reporting to the department of gynaecology with the complaint of dryness of vagina, hot flushes and palpitations. They were subjected to a questionnaire for evaluation of their oral dryness. Recruitment in the study was based on the positive response of the patients to any of the questionnaire for oral dryness and comprised the study group. The xerostomia questionnaire used to evaluate oral dryness was proposed by Torres et al. [18] using the modified eight-item version by Nunes et al. [19, 20]. It composed of 8 questions related to xerostomia. Control group comprised of post menopausal females with no complain of oral dryness and with negative response to all the questionnaires related with oral dryness.

### Characterization of study sample

The study was conducted as per the guidelines of Helsinki declaration. Ethical clearance of the research was taken from the institutional review board of Shijiazhuang Maternity Hospital with approval number 20210977H970 and written informed consent was also obtained from the respective subjects before enrolment in the study sample. All the enrolled subjects were subjected to a detailed case history. Only post menopausal women that too without any history of deleterious habits such as smoking, chewing tobacco and alcohol were included. The subjects were categorized as postmenopausal if there was no menstrual cycle for the past 1 year so as to reduce the bias of faulty enrolment. None of the patients were hysterectomised. The patients who were subjected to any kind of hormone replacement therapy were excluded. The patients suffering from any systemic disease, any oral disease or lesion and using any medication affecting salivary flow were also excluded. Patients with history of blood clots, stroke, heart attack, liver problems, or bleeding problems were also excluded. Oral dryness was evaluated by two authors (LW and LZ) after collection of the data on age.

#### Sample size evaluation

Sample size was evaluated with the help of OpenEpi Version 3 software. A pilot study was conducted with 5 patients in each case group and control group as per the inclusion and exclusion criteria. The mean difference of eastradiol levels before and after HRT in case group in these 5 patients at 95% CI was 1.32 ± 0.95 which revealed a minimum sample size for the case group to be 20 subjects.

However a total of 30 patients who responded ‘Yes’ to even one of the questions of the provided questionnaire during the last 6 months duration were enrolled in the study group. A total of 30 patients who gave ‘NO’ response to all the questions of the questionnaire and were asymptomatic for at least last 6 months comprised of control group.

The patients of case group were evaluated by gynaecologists (LW and YR). The evaluation involved breast and cervical examination along with Pap smear investigation to rule out breast or cervical cancer. If these investigations were normal, the patients were subjected to HRT which constituted of conjugated equine estrogen (Tab Premarin) and progesterone (Tab Modus). Patients with salivary estrogen levels less than 0.5 pg/ml were subjected to regime I for 3–6 months while those with salivary estrogen level more than 0.5 pg/ml were given regime II for 3 months (Table 1). Periodical re-evaluation of the patients was done to determine the recommendation of further treatment. Patients were also re-evaluated periodically to determine if treatment for symptoms was still necessary. Follow up assessment of salivary estradiaol and questionnaire evaluation for oral dryness was again performed after 6 months with the same method.

**Table 1:** Regimen of HRT

Regime	Dose
Regimen I	Low dose Estrogen Tab Premarin 0.3 mg/day In cyclic regimen (25 days on and 5 days off) + Sequential MPA Tab Modus 5 mg (One Tab daily for 14 days)
Regimen II	Moderate Dose Estrogen Tab Premarin 0.625 mg/day + Sequential MPA Tab Modus 5–10 mg (One Tab daily for 14 days)

#### Saliva sample collection

To maintain consistency as well as suitability of the saliva sample for precise qualitative analysis, all the patients were advised to avoid foods with high sugar or caffeine products immediately before the salivary sample collection. They were also instructed to rinse their mouth with distilled water and relax for at least 15 min before saliva collection. No subject was allowed to brush the teeth, eat any meal or drink anything for at least one hour before saliva collection. Later unstimulated saliva was collected by passive drool method. The saliva was collected by the patients in their mouth and subsequently the head was tiled forward. They were instructed not to speak and swallow the saliva during this period. All the collected saliva was drained into a plastic container which was later stored at −20 degree celsius and was used to assess salivary estradiaol level by ELISA technique using salivary estradiaol kit (by DRG Instruments, Germany**)**. Consistency in collection method was maintained to avoid unsystematic error into the study data.

### Specimen preparation

The samples were brought to room temperature and centrifuged for 5–10 min producing a colourless supernatant which was pipetted out and salivary analysis was done as per the manufacturer’s instructions.

### Statistical evaluation

The statistical evaluation was done using statistical package for social sciences (Inc, Chicago, IL) version 16.0. Student’s unpaired ‘t’ test evaluated the difference in mean ages and inter group differences of means. Paired ‘t’ test evaluated intragroup variation of mean values. Chi Square test was used to compare the response of patients towards the questions.

## Results

The mean age of patients of case group was 55.60 ± 5.29 years while the mean age of control group was 53.87 ± 3.94 years. The mean age was compared using unpaired t-test. There was no significant difference in mean age between case and control groups with *p* value of 0.155 (t-test value 1.440).

The mean salivary estradiol levels in case group and control group before HRT is shown in Table 2. Unpaired t test reveals that the mean salivary estrogen level before HRT was significantly more among control group compared to case group (*p* value < 0.001).

**Table 2:** Salivary estrogen level before HRT

	Mean	SD	Mean difference	t-test value	*p*- Value
Case	0.97	0.73	− 3.55	− 13.131	< 0.001*
Control	4.52	1.29			

*Significant difference (Unpaired t-test)

The mean salivary estrogen level was compared before and after HRT in the case group using the paired t-test. The mean salivary estrogen level increased significantly after HRT (*p* value < 0.001) (Table 3).

**Table 3:** Comparison of salivary estrogen level in the case group before and after HRT

Salivary estrogen level	Mean	SD	Mean difference	t-test value	*p* Value
Before HRT	0.97	0.73	− 1.36	− 11.149	< 0.001*
After HRT	2.34	1.05			

Paired t-test, *Significant difference

The distribution of question 1–8 was compared before and after HRT in the case group using the chi-square test (Table 4). Feeling of dry mouth during meals, having difficulty swallowing food, perceive small amount of saliva in your mouth most of the time, feeling of dry mouth at night or upon waking and feeling of dry mouth during the day decreased significantly from before HRT to after HRT.

**Table 4:** Response to questionnaires before and after HRT in the case group

		Before HRT		After HRT		Chi-square value	*p* Value
		N	%	N	%		
Question 1: Feel dry mouth during meals	No	4	13.3	18	60.0	14.067	< 0.001*
	Yes	26	86.7	12	40.0		
Question 2: Have difficulty in swallowing food	No	15	50.0	24	80.0	5.934	0.015*
	Yes	15	50.0	6	20.0		
Question 3: Perceive small amount of saliva in your mouth most of the time	No	5	16.7	22	73.3	19.461	< 0.001*
	Yes	25	83.3	8	26.7		
Question 4:Feel dry mouth at night or upon waking	No	2	6.7	17	56.7	17.330	< 0.001*
	Yes	28	93.3	13	43.3		
Question 5:Feel dry mouth during the day	No	5	16.7	15	50.0	7.500	0.006*
	Yes	25	83.3	15	50.0		
Question 6:Chew gum or mints to relieve the sensation of dry mouth	No	23	76.7	26	86.7	1.002	0.318
	Yes	7	23.3	4	13.3		
Question 7:Frequently wake up thirsty at night	No	24	80.0	27	90.0	1.176	0.278
	Yes	6	20.0	3	10.0		
Question 8: Have a burning sensation on your tongue	No	28	93.3	28	93.3	< 0.001*	1.000
	Yes	2	6.7	2	6.7		

Chi-square test, *Significant difference

## Discussion

Estrogen is an ovarian sex hormone and is responsible for secondary sexual characteristics in females [21]. Out of the type of estrogens, beta-estradiol or estradiol is most important and is primarily secreted by ovaries [21]. It regulates many functions in the body including its effects on the female sex organs, fallopian tubes, breasts, skeleton and metabolism of proteins and fat. Hence it is considered crucial for the maintenance of health of women. However there is evidence of reduction of estradiaol levels in postmenopausal females which can manifest as systemic or oral effects. Its immediate systemic effect includes hot flushes, swelling, palpitation, dysurea, recurrent urinary tract infection (UTI), mood disturbance and reduced libido. Long term systemic effects include osteoporosis, cardiovascular disturbance and dementia to name a few [1–3].

Oral effects due to declined estrogen in postmenopausal females include xerostomia. It is also known as hyposialia or dryness of mouth [1, 22–24]. Researchers attribute this dryness of mouth to increased immunoglobulin A (IgA) and total protein [22]. This can further initiate oral discomfort, root caries, taste alteration, periodontal (PDL) disease and oral candidiasis in such women [22–26]. Henceforth the altered estrogen level in postmenopausal women can lead to significant alterations within the oral cavity.

A study conducted by Wardrope and co-workers revealed that oral discomfort was evident in 33% of the postmenopausal women and was significantly higher than premenopausal females [1, 23]. It was of interest to note that Minicucci et al. [5] revealed reduction in salivary flow in menopausal females, however there was no symptom present in them related to xerostomia. Similarly Saluja et al. in the year 2014 revealed reduced salivery pH as well as taste perception by postmenopausal females as compared to other group [1, 27].

Although the effect of menopause on oral cavity is widely discussed in literature, the effect of menopause in consideration to oral dryness related to estrogen levels in saliva has received limited attention. Furthermore the effect of hormone replacement therapy on estrogen levels and oral dryness is limited [1, 14, 28–33]. The literature regarding these studies is scarce and the effect of hormone replacement therapy in recovering the estradiaol levels in saliva is not reported so far. This study reveals that there exist symptoms of xerostomia in postmenopausal females but most of the time the women are unaware of this feeling. Further the levels of salivary estradiol are significantly less in those postmenopausal females who gave positive response to any one of the questionnaires related to xerostomia. It can be attributed to the fact that this feeling is subjective unless there is any other symptom of oral discomfort evident. It can also be supported by the fact that some postmenopausal women reveal decrease in salivary flow rate while some even reveal increase or even no change in the salivary flow rate [14, 28–33].

Certain researchers like Valima et al. [34] revealed that the estrogen receptor can be located on the oral mucosa, gingiva and salivary glands. Leimoli-Virtanen et al. in 1997 revealed that salivary composition as well as salivary flow are dependent upon estrogen [35]. Henceforth some of the females were totally relieved by their symptoms and they did not reported ‘yes’ to any of the questions of the questionnaire. In this study, feeling of dryness of the mouth was most common symptom experienced among the case group. This fact was in accordance to the study conducted by Forabosco et al. [13]. This could be attributed to the fact that there can be qualitative changes in the composition of saliva due to reduced estrogen. It can also be due to alteration in the mucosal sensory receptors [13]. This finding was also supported by Wardropa et al. [23] and Ferguson et al. [28].

As discussed earlier, several authors like Wardropa et al. [23] and Forabosco et al. [28] revealed that postmenopausal females were relieved of the feeling of oral discomfort after hormone replacement therapy. It was also in accordance to the results of this study. However it is of interest to note that this study reveals recovery of the salivary levels of estradiol after females were subjected to hormone replacement therapy. Consequently the corresponding symptoms of xerotomia also reduced. This study utilised unstimulated saliva sample. This was in accordance to the study conducted by Bergdahl in the year 2000 [14, 36] which reveals that this method is more reliable for the cases of hyposalivation. This was in contrast to few other studies where stimulated saliva was used [36]. Since hormone replacement therapy restores the salivary flow rate, it was assumed that probably estradiol recovery was also possible. Henceforth estradiol was evaluated.

Conjugated estrogen was given to restore the estrogen levels. Tablet Modus was given to prevent endometrial hyperplasia associated with estrogen therapy. Since HRT have certain side effects, it must be prescribed after proper evaluation. HRT was indicated in the case group patients because all the included patients of case group were suffering from genitourinary symptoms like vaginal dryness and hot flushes with reduced estrogen levels [37]. Further the case group of our study also presented with oral dryness apart of having regular vaginal dryness and hot flushes. Henceforth systemic HRT was indicated under the supervision of a gynaecologist with proper regimen [38]. To reduce the chances of any side effect, a robust exclusion criterion was followed as mentioned in the methodology.

As far as the questionnaire was concerned, the patients reported improvement in most of the symptoms after HRT (Table 4). However it is of interest to note that HRT was not able to improve the subjective feeling of chewing gum or mints to relieve the sensation of dry mouth (*p* value 0.138). It was even not able to relieve the symptom related to waking up thirsty at night (*p* value 0.278) and burning sensation on tongue of the patients (*p* value 1.0).

Since literature reveals that salivary flow rate may not change significantly during menopause [29], the levels of estradiol could have been possibly the reason behind the feeling of oral discomfort. It was in contrast to the study of Giusti et al. [14] which reveals that salivary flow was significantly reduced during the Menopause and it was recovered by hormone replacement therapy. However smaller sample size was a limitation of this study.

Future multicentre studies can better target this research question. Moreover longer follow up would be more beneficial in assessing long term resolution of oral dryness in such females. This study was based on the subjective responses to the oral dryness questionnaire. Although minimal but it could have been a reason for reporting bias. Henceforth we considered ‘Yes’ or ‘No’ response to even a single questionnaire sufficient enough to include or exclude a participant so as to reduce this bias. Estimating the salivary flow would have been a better parameter to consider removing this potential bias.

## Conclusion

Postmenopausal females with symptoms of oral dryness can present with reduced salivary estradiaol levels. Secondly, HRT can be effective in the management of such patients in terms of recovery of salivary estradiaol and subsequently relieving symptoms of oral dryness. Future multicentre studies can be more beneficial in this context keeping in mind the side effects of the therapy.

## Data Availability

The datasets used and/or analyzed during the study are available from the corresponding author on reasonable request.

## Abbreviations

ELISA: Enzyme linked immuno sorbant assay
HRT: Hormone replacement therapy
WHO: World Health Organization
UTI: Urinary tract infection
CVS: Cardio vascular system
PDL: Periodontal ligament
